# Dr. Arthur B. Carpenter

**Published:** 1890-12

**Authors:** 


Dr. Arthur B. Carpenter, of Cleveland, O., died suddenly in that city recently. He was a rising young physician, and gave promise of a successful future. His remains, in accordance with his previously expressed wish, were incinerated at the Buffalo Crematory, October 17, 1890.

